# Doctors

**Published:** 1844-07

**Authors:** 


*Doctors.*—Now that I am talking of doctors, what a strange set they are, and what a singular position they hold in society! Admitted to the fullest confidence of the world, yet by a strange perversion, while they are the depositories of secrets that hold together the whole fabric of society, their influence is neither fully recognized, nor their power acknowledged.

The doctor is now, what the monk once was, with this additional advantage, that from the nature of his studies, and the research of his art, he reads more deeply in the human heart, and penetrates into its inmost recesses. For him, life has little romance; the grosser agency of the body, reacting ever on the operations of the mind, destroys many a poetic daydream and many a high-wrought illusion. To him alone does a man speak, "*son dernier mot*," while to the lawyer, the leanings of self-respect will make him always impart a favorable view of his case. To the physician he will be candid, and even more than candid; yes, these are the men, who, watching the secret workings of human passions, can trace the progress of mankind in virtue and in vice; while ministering to the body they are exploring the mind; and yet scarcely is the shadow of fear dissipated, when they fall back to their humble position in life, bearing with them little gratitude; and, strange to say, no fear!

The world expects them to be learned, well bred, kind, considerate, and attentive, patient to their querulousness, and enduring under their caprice; and after all this, the humbug homœpathy, the preposterous absurdity of the water cure, or the more reprehensible mischief of mesmerism, will find more favor in their sight than the highest order of ability accompanied by great natural advantages.

Every man, and still more, every woman imagine themselves to be doctors. The taste for physic, like that for politics, is born with us, and nothing seems easier than to repair the injuries of the constitution, whether of the state or individual. Who has not seen, over and over again, physicians of the first eminence put aside, that the nostrum of some ignorant pretender of the suggestive twaddling old woman, should be, as it is termed, tried? No one is too stupid, no one too old, no one too ignorant, too obstinate, or too silly, not to be superior to Brodie and Chambers, Crampton and Marsh; and where science, with anxious eye and cautious hand, would scarcely venture to interfere, heroic ignorance would dash boldly forward and cut the gordian difficulty by snapping the thread of his life. How comes it that these old ladies of either sex never meddle with the law? Is the game beneath them, when the stake is only property and not life? or is there less difficulty in the knowledge of an art whose principles rest on so many branches of science, than in a study founded on the basis of a precedent? Would to heaven the "ladies bountiful" would take to the quarter sessions and the assizes, in lieu of the infirmaries and dispensaries, and make Blackstone their aid-de-camp, vice Buchan retired.—*Dublin University Magazine.*

